# TaiChi-MSS protocol: enhancing cognitive and brain function in MCI patients through Tai Chi exercise combined with multisensory stimulation

**DOI:** 10.3389/fnagi.2025.1514127

**Published:** 2025-02-25

**Authors:** Chunhui Zhou, Ganfeng Yang, Yinying Wang, Ruiting Zhu, Dong Zhu

**Affiliations:** ^1^School of Wushu, Shanghai University of Sport, Shanghai, China; ^2^Physical Education Institute, Soochow University, Suzhou, Jiangsu, China

**Keywords:** mild cognitive impairment (MCI), sensory function, Mini-Mental State Examination, clinical dementia rating, Montreal Cognitive Assessment, brain activation, domain-specific cognitive function

## Abstract

**Background:**

The aging population in China is confronted with considerable challenges, with 14.71% of elderly individuals affected by mild cognitive impairment (MCI). The practice of Tai Chi has been demonstrated to enhance cognitive function, while sensory stimulation has been shown to facilitate neural activity. Nevertheless, the combined impact of Tai Chi and sensory stimulation on cognitive, sensory functions, and brain activation in older adults with MCI remains uncertain. This study aims to ascertain whether the integration of Tai Chi with sensory stimulation can facilitate more efficacious interventions for these outcomes.

**Methods and analysis:**

The TaiChi-MSS (Tai Chi and Multisensory Stimulation for Cognitive Function) study is a multi-center, randomized controlled trial (RCT) conducted in Suzhou and Shanghai, enrolling 88 participants aged 60 years or older with MCI. Participants will be randomly assigned to one of four groups: Tai Chi, multisensory stimulation, Tai Chi combined with multisensory stimulation or control. The intervention will last 6 months, with follow-up assessments at 3, 6, and 9 months. Primary outcomes include cognitive and sensory assessments, assessed using the Montreal Cognitive Assessment (MoCA), Mini-Mental State Examination (MMSE), domain-specific cognitive tests, Pure Tone Audiometry (PTA), and Sniffin’ Sticks Odor Identification Test. Secondary outcomes involve brain activation, measured through functional Magnetic Resonance Imaging (fMRI) scans. fMRI will be used to assess brain structure and connectivity changes, focusing on neuroplasticity. Data will be analyzed using mixed-effects models. The False Discovery Rate (FDR) will be the correction method for multiple comparisons to control for the expected proportion of false positives.

**Ethics and dissemination:**

This study was approved by the ethics committee of Shanghai University of Sport (No. 102772023RT200). The results of this study will be disseminated in peer-reviewed journals and presented at academic conferences.

## Introduction

China is one of the greatest countries that undergoes worldwide and rapid demographic progress, with the proportion of people aged 65 reaching 12.6% of the total population. In urban areas such as Shanghai, this proportion has already reached 21.8%, making it a “super-aged” city (Mitchell, 2015). This demographic shift presents a significant challenge, particularly with regard to the cognitive decline associated with aging. Alzheimer’s disease (AD), the most prevalent form of dementia, is typified by progressive cognitive decline, loss of functional autonomy, and considerable societal and economic burdens (Jack et al., 2024).

The findings of recent research indicate that Alzheimer’s disease commences its development approximately 10–15 years prior to the point at which a clinical diagnosis is made. This evidence underscores the vital importance of early intervention (Amieva et al., 2008; Bateman et al., 2012). Mild cognitive impairment (MCI) has become a key focus for preventive strategies, as it represents a transitional stage between normal cognitive aging and dementia (Jin et al., 2023; Rozzini et al., 2007). MCI is characterized by subjective cognitive complaints and measurable cognitive impairment, though functional abilities remain largely intact and do not yet meet the criteria for dementia (Anand and Schoo, 2024). In China, approximately 14.71% of the elderly population is estimated to be affected by MCI, underscoring the urgent need for effective intervention strategies (Xue et al., 2018). Especially at early preclinical stages such as early and stable MCIs (Zhou, 2021), these advancements hold significant promise for improving early diagnosis and intervention strategies.

In 2021, the US Food and Drug Administration granted accelerated approval to aducanumab for patients with MCI and mild dementia caused by Alzheimer’s disease (AD) (Terao et al., 2022). However, aducanumab was less well-tolerated than placebo, and its clinical outcomes did not meet expectations (Terao and Kodama, 2024). As a result, there has been a shift in focus toward non-pharmacological interventions. Among these, exercise, particularly aerobic and resistance training, has shown the potential to improve cognitive functions such as memory, executive function, and attention (Abrahamson et al., 2016; Cammisuli et al., 2017; Chow et al., 2021; Law et al., 2020; Loprinzi et al., 2019; Park et al., 2019; Song and Yu, 2019; Young et al., 2015; Zheng et al., 2016; Zhou, 2019). However, some studies indicate that the effects of these interventions on executive function and episodic memory are inconsistent (Ballesteros et al., 2024).

Tai chi emphasizes the coordination of the body, breathing, and mind, and as a potentially effective exercise method to improve brain health and slow down brain aging, Tai chi is attracting increasing attention (Loprinzi et al., 2013). Studies have shown that Tai Chi improves memory and executive function in individuals with MCI, potentially through mechanisms such as the increased expression of brain-derived neurotrophic factor (BDNF), which promotes neuroplasticity (Jasim et al., 2023; Zhang et al., 2023). However, despite growing interest, the exact mechanisms by which Tai Chi affects brain structure and function remain unclear, and further large-scale, long-term trials are necessary to confirm these effects (Miller and Taylor-Piliae, 2018; Wayne et al., 2018).

Recent studies have also explored the potential of sensory stimulation as an adjunct therapy for cognitive enhancement (Dimitriou and Tsolaki, 2017; Lykkeslet et al., 2014; Yang et al., 2021). Engaging multiple sensory modalities—such as vision, hearing, and smell, is hypothesized to enhance neural activity and improve cognitive function in patients with neurodegenerative conditions (Glachet and El Haj, 2020; Yang et al., 2021; Yoshii et al., 2019). Although promising, there is limited clinical evidence on the combined effects of Tai Chi and MSS on both cognitive and sensory function (Zhi-lei and Zhu, 2018). Moreover, the relationship between sensory decline and cognitive deterioration in MCI populations remains underexplored (Deal et al., 2016; Sayyid et al., 2024).

Thus, this study aims to investigate the effects of Tai Chi combined with multisensory stimulation on cognitive function, brain structure, and sensory function in older adults with MCI. We hypothesize that this combined intervention will lead to superior improvements in both cognitive and sensory outcomes compared to single-modality interventions, potentially through the “sensory-brain-cognitive” pathway. This research aims to provide critical insights into novel non-pharmacological interventions for MCI, contributing to the development of effective and scalable prevention strategies for dementia.

## Methods and analysis

### Objectives and study design

The TaiChi-MSS study is a randomized, controlled, single-blind trial conducted across multiple centers in Suzhou and Shanghai, in which the outcome assessors will be blinded to group assignments. Block randomization, with a fixed block size of four, will be used to allocate enrolled participants into one of four arms, ensuring balanced group allocation. A total of 22 participants will be assigned to each arm, with random allocation to the Tai Chi group (TCG), the Tai Chi combined with multisensory stimulation group (TMCSSG), the control group (CG), or the multisensory stimulation group (MSSG) according to a 1:1:1:1 allocation ratio. Outcome assessors will be masked to allocate and will not be involved in the interventions. Primary outcomes include assessing changes in cognitive function—such as attention, memory, language, visuospatial ability, and executive function—after a 24 weeks intervention period. Secondary outcomes involve evaluating sensory function through air conduction, pure tone hearing thresholds, and olfactory recognition levels, as well as analyzing the correlation between hippocampal subregion atrophy rates and volumes of auditory and olfactory regions using structural MRI.

**FIGURE 1:**
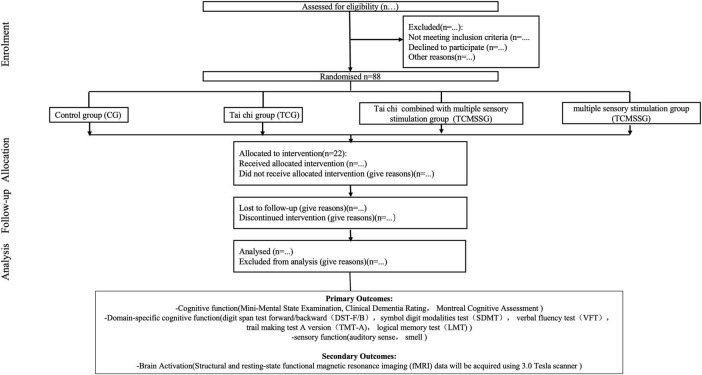
Trial flowchart.

As illustrated in Figure 1, this study will be conducted in three phases. Phase 1 is the development of the intervention, and Phase 2 comprises the screening, baseline assessment, and randomization of the participants and the execution of the multidomain intervention. Phase 3 is the approach modeling.

### Sample size

Sample size calculation: The sample size was estimated using G*Power software, incorporating considerations for cognitive and fMRI outcomes. Adjustments were made to account for dropout rates, multiple comparisons, and desired statistical power.

For cognitive outcomes, the required sample size was calculated based on an effect size of, derived from previous studies evaluating Tai Chi interventions in older adults with MCI (Bossers et al., 2015). Similar studies report comparable effect sizes regarding improvements in cognitive function (Guo et al., 2024). To achieve 95% power at a significance level, a total of 56 participants are required. Accounting for an anticipated dropout rate of 35% (Dignan et al., 2016), the adjusted sample size is approximately 88 participants. This recruitment target considers potential challenges such as health issues or non-adherence, and measures like flexible scheduling, regular follow-ups, and participant engagement will be implemented to minimize dropout.

For the fMRI analyses, an effect size was selected based on prior research exploring brain activation and connectivity changes resulting from physical and cognitive interventions (Li et al., 2012; Vance et al., 2024). To achieve sufficient power, 36 participants are required. Considering a 20% dropout rate, the adjusted sample size is 44 participants. Participants who discontinue the fMRI assessments will remain included in the cognitive analysis to ensure comprehensive data utilization.

### Criteria for participants’ inclusion and exclusion

The study started on 1 May 2024, and patients with MCI who lived in Shanghai and Suzhou communities were recruited. First, a geriatrician checked the following initial eligibility criteria: age 60 years or older, diagnosis of dementia reported in the patient’s medical file by the dementia diagnosis team or a medical specialist, and absence of serious health problems that could preclude safe participation (Bossers et al., 2015). Next, a trained Human Movement Sciences (HMS) research assistant tested the patients for the following additional inclusion criteria: Mini-Mental State Exam (MMSE) score of at least 19 to no more than 23 and the ability to perform the timed up-and-go test (Podsiadlo and Richardson, 2015), ADL score above 26 (Fuentes et al., 2020). Those who passed these six criteria completed a neuropsychological test and fMRI at baseline (time zero, T0) administered by an experienced and trained research assistant.

Exclusion criteria include participants with severe medical conditions (e.g., advanced cardiovascular disease, uncontrolled diabetes, cancer) or neurodegenerative disorders (e.g., Huntington’s disease, Parkinson’s disease) to ensure the study focuses on individuals with MCI. Participants with exercise limitations (e.g., recent surgery, joint issues), those unable to undergo MRI due to contraindications (e.g., pacemakers, metal implants, claustrophobia), or those with a history of excessive alcohol use (> 14 drinks/week) or smoking (> 10 cigarettes/day) will also be excluded to minimize confounding factors.

### Baseline measurement

At baseline, comprehensive information will be collected on participants’ sociodemographic characteristics, medical history, lifestyle factors, and anthropometric and body composition measurements. In addition, psychological and cognitive assessments will be conducted to evaluate baseline cognitive function and mental health status.

Cognitive function will be assessed using validated tools, including the Montreal Cognitive Assessment (MoCA) and the Mini-Mental State Examination (MMSE), to measure general cognitive abilities. Additionally, domain-specific cognitive functions will be evaluated using tests such as the digit span test forward/backward (DST-F/B), symbol digit modalities test (SDMT), verbal fluency test (VFT), trail-making test A version (TMT-A), and logical memory test (LMT). Sensory function will be assessed with tools like the Pure Tone Audiometry (PTA) and the Sniffin’ Sticks Odor Identification Test. For a comprehensive summary of these outcomes and assessments, please refer to Table 1. The assessments above will be conducted by trained research personnel. All personnel have undergone extensive training to ensure the reliability of the testing process.

**TABLE 1:** The parameters collected at baseline.

	Parameters
**Primary outcomes**	
Cognitive function	Mini-Mental State Examination (MMSE), Montreal Cognitive Assessment (MOCA)
Domain-specific cognitive function	Digit span test forward/backward (DST-F/B), symbol digit modalities test (SDMT), verbal fluency test (VFT), trail making test A version (TMT-A), logical memory test (LMT)
sensory function	Pure Tone Audiometry (PTA), Sniffin’ Sticks Odor Identification Test
**Secondary outcomes**	
Brain activation	Structural, and resting state functional magnetic resonance imaging (fMRI) data will be acquired using 3.0 Tesla scanner

At least five participants from each group will be randomly selected to undergo structural and resting-state functional magnetic resonance imaging (fMRI) using a 3.0 Tesla scanner. Structural MRI will allow for detailed assessment of brain anatomy, focusing on changes in brain volume, particularly in regions associated with cognitive function, such as the hippocampus and prefrontal cortex. Resting-state fMRI will assess brain connectivity and activation patterns without the need for task-based stimuli, making it ideal for observing intrinsic brain network activity (Dickerson and Sperling, 2008). The baseline scans will establish a reference point for each participant’s brain structure and function, while the post-intervention scans will reveal how the multi-domain intervention influences brain changes over time.

As part of the baseline characteristics assessment, participants will receive written feedback on their medical and health parameters, which include blood pressure (systolic and diastolic), blood glucose (fasting or random), body mass index (BMI), heart rate (HR), oxygen saturation (SpO_2_), and basic physical activity level (assessed through a questionnaire or pedometer). Participants will be advised to consult their doctor for appropriate medical management if any concerns are identified, such as high blood pressure, hyperglycemia, abnormal BMI, or low oxygen saturation.

### Intervention program

A factorial experimental design with four factors 4 × 4 will be used. All participants will be randomly assigned to one of the four groups: control group (CG), Tai Chi group (TCG), multisensory stimulation group (MSSG), or Tai Chi combined with multisensory stimulation group (TCMSSG). Key characteristics will be stratified; the stratification factors considered in this study include sex, years of education, and age to ensure that the groups are balanced.

The intervention will last for 6 months, followed by assessments at baseline, 3, 6, and 9 months. These assessments will include changes in overall cognitive function, measured through attention, memory, language, visuospatial ability, and executive function. Sensory function will be evaluated using air conduction, pure tone hearing threshold, and olfactory recognition tests. Functional MRI (fMRI) will be utilized to evaluate neural network connectivity and the effectiveness of the intervention on brain function.

The intervention for the Tai Chi groups (TCG and TCMSSG) will be based on exercise recommendations for older adults from the American College of Sports Medicine (ACSM). The 6 months intervention will include three weekly sessions, each lasting 50 min. The target heart rate (THR) will be calculated using the reserve heart rate percentage method (Table 2):

**TABLE 2:** Exercise intensity intervention program [Tai Chi group (TCG) and Tai Chi combined with multisensory stimulation group (TCMSSG)].

Elements of intervention	Initial stage (4 weeks)	Mid-term (10 weeks)	Late stage (10 weeks)
Intensity of exercise	45% 55% HRmax	55% 65% HRmax	65% 75% HRmax
Frequency of movement	3 times a week	3 times a week	3 times a week
Duration	40 60 min	50 70 min	50 70 min


THR=(HRmax-HRrest)×intensity⁢(45%-75%)+

HRrest⁢(Lindenberger, 2008).

Additionally, the Borg Rating of Perceived Exertion (RPE) scale will be used to monitor participants’ subjective fatigue levels during the sessions.

1. Tai Chi Group (TCG): Participants will practice four sets of eight-style Tai Chi routines focused on balance, coordination, flexibility, and strength exercises. Each session will be 50 min long, with varying exercise intensities over the course of the intervention (starting at 45% HRmax and progressing to 75% HRmax). The “Eight-Style Tai Chi” mentioned in the manuscript refers to a standardized routine. This routine is designed as an introductory Tai Chi practice, consisting of eight movements. The Eight-Style Tai Chi is entirely based on the Yang-style Tai Chi and incorporates its eight most fundamental and essential movements. The routine is concise, focuses on key principles, and is characterized by its simplicity, ease of learning, and smooth, slow execution.

2. Tai Chi Combined with Multisensory Stimulation Group (TCMSSG): In addition to the Tai Chi exercises, this group will experience multisensory stimulation through soothing music and essential oils. The music volume will be controlled, and four types of essential oils (rosemary, lemon, rose, and lavender) will be used. Each essential oil will be diffused at a separate intervention site using an aroma diffuser. At each site, the essential oil will be used in four different concentrations (3, 7, 10, and 13%), progressing from low to high concentration during the intervention to address the sense of smell’s adaptation to the scent over time. Participants will rotate through four different intervention sites (Site A, Site B, Site C, and Site D), each with a specific combination of Tai Chi and sensory stimulation (e.g., Tai Chi 1 + A music + rosemary oil diffused in 3, 7, 10, and 13% concentrations). Adjustments to the concentrations will be made during the practice to ensure optimal effectiveness.

3. Control Group (CG): Participants in the control group will not undergo any exercise intervention. They will be asked to maintain their regular daily activities, which will be monitored using the International Physical Activity Questionnaire Short Form (IPAQ-SF) every 4 weeks. It will be ensured that their physical activity does not exceed 600 METs-min/week during the intervention period (Park et al., 2023).

4. Multisensory Stimulation Group (MSSG): Participants in the MSSG will receive auditory and olfactory stimulation identical to the TCMSSG but without the Tai Chi exercises. The stimulation sessions will last for 50 min each, and participants will be asked to record their emotional responses to the stimuli after each session.

### Intervention and follow-up

**FIGURE 2:**
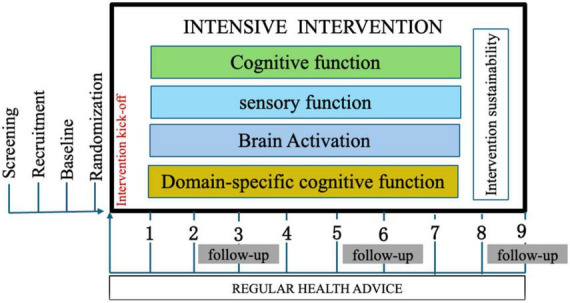
Trial protocol.

The study comprises a 6 months intervention period, followed by a 3 months extended follow-up phase (see Figure 2). Outcome measures will be assessed at the outset of the study and at 3 months intervals thereafter. Additionally, a 3 months follow-up period will be conducted to ascertain the sustainability and efficacy of the implemented intervention module. During this phase, respondents will not be required to attend any sessions with the researchers.

### Modeling methods to reduce the risk of MCI

The third phase will be based on the analysis of the first and second phases for the prevention or reversal of the MCI multi-domain intervention module. A feasibility study and implementation will be carried out in Shanghai communities. Important strategies from Phase I and Phase II will be integrated into one module to reverse MCI in the future by establishing treatment rooms for motor combined with sensory stimulation. The model will include elements and strategies for a multi-domain intervention and is primarily used by stakeholders involved in elderly care, including policymakers, public health strategists, and health and local volunteers. These applications will provide standardized protocols and easy-to-use intervention guidelines to support the prevention and management of older adults with MCI.

### Statistical analysis

Statistical Package for Social Sciences (SPSS) software (version 23.0) will be used for statistical analysis. An alpha level of (0.05) will be considered for all statistical tests. Two-sided *P*-values of (0.05) and (80%) power indicate statistical significance (Schulz et al., 2010). A mixed effect model will analyze the results and be employed for statistical analysis of the variance of the pretest (baseline score) and posttest (3, 6, 9 months) in the four groups. All mental, cognitive, sensory, and physical outcome measures will be used for statistical analysis. Discovery Rate (FDR) correction will be used for *post hoc* analysis.

Given the variety of cognitive and fMRI outcome measures, we will apply the False FDR to control for multiple comparisons (Genovese et al., 2002). Bonferroni correction will be used only if necessary for specific hypotheses with fewer comparisons, but FDR will be the primary approach (Alspach, 1996). An intention-to-treat (ITT) approach will be employed to handle missing data (Gupta, 2011; Little, 2024). Multiple imputations will be applied for missing cognitive data. Participants with incomplete or poor-quality fMRI data, such as those affected by motion artifacts, will be excluded from the fMRI analysis but kept in the cognitive analysis. Specific handling of missing data will depend on the outcome type.

Functional MRI (fMRI) will be utilized to evaluate neural network connectivity and the effectiveness of the intervention on brain function. Pearson correlation analysis will be conducted to assess the relationship between hippocampal subregion atrophy and the volumes of auditory and olfactory regions using structural MRI data. Similarly, Pearson or Spearman correlation coefficients will be calculated, depending on the distribution of the data, to explore the associations between fMRI-derived connectivity metrics and cognitive and sensory outcomes. These analyses aim to uncover potential neural mechanisms underlying the effects of the interventions. A sensitivity analysis will be performed by varying hypothetical dropout rates and effect sizes to assess their impact on the statistical power and required sample size (Little, 2024).

### Relevance to government policy

The project aligns with the “14th Five-Year” Healthy Aging of the National Health Commission. The results of this project are of great value to the development of the national old-age cause and the planning of the old-age service system.

## Ethics and dissemination

### Data management

The study will be conducted following Good Clinical Practice (GCP) guidelines. Adherence to two basic principles should be maintained: (1) Data Protection to ensure the anonymity and privacy of our participants; and (2) Data Sharing to ensure that data from publicly funded research are publicly available. We will ensure that all institutional Data Protection requirements are followed. Potentially identifiable personal data will be stored only within secure locations and electronic data in local secure servers, as stipulated within the Personal Data Protection Act 2010 (Ponvel et al., 2021). We would be anonymizing data and enlisting our institutional librarians to assist with data curation before depositing our data in an open data repository within 12 months of project completion.

All physical materials, such as consent forms, completed questionnaires, and demographic details, will be secured in a locked cabinet at the research center at Shanghai University of Sport. Participants will be assigned a numerical code separately to ensure their privacy. Participant names or other identifying features will not appear in data reporting. The coding system will be stored digitally and manually at Soochow University and accessible only to research team members.

### Ethics approval and consent to participate

This study was approved by the Scientific Research Ethics Committee of Shanghai University of Sport on 15 March 2024 (102772023RT200) and approved by the Chinese Clinical Trial Registry on 24 April 2024 (ChiCTR2400083424). All volunteers or their legal representatives will be asked to sign a written informed consent form with prior approval from the ethics committee.

### Discussion

Mild cognitive impairment (MCI) is progressive and affects memory, other cognitive abilities, and motor performance (Jia et al., 2020). These impairments can interfere with an individual’s ability to perform daily activities and are often associated with sensory impairments, which exacerbate cognitive decline (Clemmensen et al., 2020).

Research has shown that sensory disturbances can lead to decreased concentration, increased dependency, and reduced quality of life for patients with MCI (Bouhaben et al., 2024; Deal et al., 2016; Jacobsen et al., 2015). Therefore, early interventions targeting both cognitive function and sensory organ health are essential to maintaining independence and improving quality of life for this population. Tai Chi, a mind-body exercise, has the potential to enhance brain function through its focus on strength, balance, and dual-tasking (Jia et al., 2020; Ponvel et al., 2021). By combining physical movement with controlled breathing and mental focus, Tai Chi promotes both sensory integration and physical adaptation to the environment (Jasim et al., 2023). Sensory function plays a critical role in how the brain processes environmental stimuli, and enhanced sensory input, together with physical activity, may improve functional performance, slow cognitive decline, boost mood, and increase confidence, thereby reducing dependence (Drapeau et al., 2009; Liu et al., 2021; von Bornstadt et al., 2018; Won et al., 2021).

A key strength of this study compared to similar ongoing research is the integration of sensory function into the intervention. While some studies suggest that sensory decline may be associated with cognitive dysfunction, most existing research has focused on patients with Alzheimer’s disease (Jung et al., 2019). This study seeks to extend these findings by investigating the effects of combining motor and sensory interventions on cognitive function in older adults with MCI. Additionally, this study aligns with the World Health Organization (WHO) Global Action Plan for the Public Health Response to Dementia 2017–2025 and the latest Lancet Commission report on dementia prevention and care. It addresses the need for comprehensive data on dementia risk factors and explores innovative approaches to prevention, particularly within culturally and geographically similar environments to China (Ponvel et al., 2021).

This outcome will provide new insights into how sensory and cognitive interact in response to multi-domain interventions. Tai Chi, as a non-invasive intervention exercise practice and one of the traditional Chinese Medicine components, is generally beneficial for recovery of brain cognitive function and rehabilitation and has also been applied extensively in other diseases such as traumatic brain injury (TBI) cases (Zhou, 2020). Multi-domain interventions, such as the one employed in this study, have been suggested as potential strategies to reduce healthcare costs associated with aging populations (Reparaz-Escudero et al., 2024). This study introduces a novel approach by integrating sensory stimulation with Tai Chi to target cognitive and sensory decline in older adults with MCI. Findings are expected to inform public health strategies, contribute to aging policies, and provide a model for scalable, sustainable interventions in similar cultural and demographic contexts.

Future directions for this research could include conducting a detailed correlation analysis between neuropsychological assessments and neuroimaging data. Specifically, investigating the relationship between sensory processing regions and memory-related brain areas, as well as examining the correlation between cognitive scores and specific brain regions, could provide a deeper understanding of the neurobiological mechanisms underlying the observed cognitive improvements. Additionally, longitudinal follow-ups designed to explore causality will be essential in determining whether multi-domain interventions lead to sustained cognitive and sensory enhancements over lasatime.

### Conclusion

This study introduces a novel approach by integrating sensory stimulation with Tai Chi to target cognitive and sensory decline in older adults with MCI. Findings are expected to inform public health strategies, contribute to aging policies, and provide a model for scalable, sustainable interventions in similar cultural and demographic contexts.
